# Early clinical experience with volumetric modulated arc therapy in head and neck cancer patients

**DOI:** 10.1186/1748-717X-5-93

**Published:** 2010-10-15

**Authors:** Marta Scorsetti, Antonella Fogliata, Simona Castiglioni, Caterina Bressi, Mario Bignardi, Piera Navarria, Pietro Mancosu, Luca Cozzi, Sara Pentimalli, Filippo Alongi, Armando Santoro

**Affiliations:** 1 Istituto Clinico Humanitas IRCCS, Radiation Oncology Dept, Milan (Rozzano), Italy; 2 Oncology Institute of Southern Switzerland, Medical Physics Unit, Bellinzona, Switzerland

## Abstract

**Background:**

To report about early clinical experience in radiation treatment of head and neck cancer of different sites and histology by volumetric modulated arcs with the RapidArc technology.

**Methods:**

During 2009, 45 patients were treated at Istituto Clinico Humanitas with RapidArc (28 males and 17 females, median age 65 years). Of these, 78% received concomitant chemotherapy. Thirty-six patients were treated as exclusive curative intent (group A), three as postoperative curative intent (group B) and six with sinonasal tumours (group C). Dose prescription was at Planning Target Volumes (PTV) with simultaneous integrated boost: 54.45Gy and 69.96Gy in 33 fractions (group A); 54.45Gy and 66Gy in 33 fractions (group B) and 55Gy in 25 fractions (group C).

**Results:**

Concerning planning optimization strategies and constraints, as per PTV coverage, for all groups, D_98% _> 95% and V_95% _> 99%. As regards organs at risk, all planning objectives were respected, and this was correlated with observed acute toxicity rates. Only 28% of patients experienced G3 mucositis, 14% G3 dermitis 44% had G2 dysphagia. Nobody required feeding tubes to be placed during treatment. Acute toxicity is also related to chemotherapy. Two patients interrupted the course of radiotherapy because of a quick worsening of general clinical condition.

**Conclusions:**

These preliminary results stated that volumetric modulated arc therapy in locally advanced head and neck cancers is feasible and effective, with acceptable toxicities.

## Introduction

Radiotherapy (RT), with or without chemotherapy, is the primary treatment modality for head and neck cancer patients. In the last decade intensity modulated radiotherapy (IMRT) has gradually assumed a wide role in the management of such diseases. IMRT has the advantage, over the previously used conformal therapy, of improving normal tissue and organ sparing together with good target coverage. The clear dosimetric benefits were translated to better clinical results in terms of reduction of toxicity, which can improve the quality of life of patient receiving RT, without compromising the probability of tumour control.

Reviews for treatment outcome and major toxicity patterns can be found in Gregoire *et al *(1), Lee *et al *(2) and in Popovtzer *et al *(3) and in references therein. On the toxicity side, besides the attention given to spinal cord and brain stem (with toxicity thresholds of 45-50 Gy for the first and at 50 Gy for the second in most of the investigations), it is consolidated knowledge that, for parotids, mean doses inferior to 25-30 Gy correlate well with substantial recovery of function within two years (Li *et al *(4), Deasy *et al *(5)). Higher thresholds were observed for sub-mandibular glands in the range of 39 Gy by Murdoc *et al *(6), while a dose to oral cavity of about 30Gy for late mucositis was reported by Narayan *et al *(7). Recently, the wide application of IMRT allowed also investigations on strategies to reduce other common toxicity patterns. As an example, the reduction of dysphagia was correlated by Feng *et al *(8) and Levendag *et al *(9) with the irradiation of the swallowing structures as the constrictor muscles.

The same high treatment quality is achievable today with other treatment techniques, as the rather new volumetric modulated arc therapy. One of these solutions is the RapidArc^(r) ^implementation (Varian Medical System, Palo Alto, CA, USA). Based on the original investigation of K. Otto (10), RapidArc^(r) ^was recently introduced in clinical practice in several institutes after an intensive validation at planning level where it was compared to IMRT or other approaches, in a series of studies on various indications (11-20).

RapidArc was also explored for head and neck patients (19-21) demonstrating a dosimetric improvement with respect to the most commonly used IMRT for organs at risk sparing, especially when using two arcs.

At the Istituto Clinico Humanitas, since January 2009, all head and neck patients are treated with RapidArc technology, generally associated with chemotherapy. Aim of the present study is to evaluate the initial clinical experience with head and neck RapidArc patients, in terms of dosimetric analysis and acute toxicity results.

## Methods and materials

### Patients' selection

This is a single-Institute non randomised retrospective study. Between January and December 2009, 45 patients presenting head and neck tumours, were treated with RapidArc at Istituto Clinico Humanitas. Table 1 shows the descriptive data of the group of patients; this is not a homogeneous cohort, indicating that the aim of the study is to report about early experiences in head and neck with RapidArc, not focussing on specific outcome or toxicity in single subgroups. It includes 28 male and 17 female with a median age of 65 years (range: 28-96 years). The primary sites of disease were oropharynx, larynx and oral cavity. Eleven patients presented a histological type different from squamous cell carcinoma (SCC). Of the SCC patients, 5 presented with stage III, 27 with stage IVA, 1 with stage IVB. Six SCC patients presented a T1/T2 stage and 27 a T3/T4 stage. Considering N parameter in the SCC group, one patient was N0, six patients showed N1 stage, 25 patients showed N2, one patient presented N3. None showed distant metastases. Each patient underwent a pre-treatment evaluation, including a complete history and physical examination, magnetic resonance imaging of head and neck region, direct flexible fibre optic endoscopic examination, chest X-ray or thoracic computed tomography (CT). Positron emission tomography (18-FDG-PET) scans were performed in 6 patients.

**Table 1:** Summary of patients characteristics at treatment start

Number of patients		45
Site	Oral Cavity Nasopharynx Oropharynx Hypopharynx Larynx Nasal Cavity and Paranasal Sinuses Other	7 3 16 1 10 6 2
Histology	Squamous cell carcinoma Differentiated carcinoma Undifferentiated carcinoma Adenoidocistic carcinoma Estesioneuroblastoma Sarcomas	34 1 2 3 3 2
Sex	Males Females	28 17
Age Performance Status	Median [range] PS 0 PS 1 PS 2	65 [28, 96] y.o. 28 11 6
Diagnostic imaging	PET RM	6 45
Stage	II III IV	4 8 33
Chemotherapy	No ChT CDDP Cetuximab	10 16 19
Radiation Dose Prescription	Group A: 69.96/54.45Gy in 33 fractions Group B: 66.0/54.45 Gy in 33 fractions Group C: 55Gy in 25 fractions	36 3 6

Patients were stratified into three groups:

- Group A: 36 patients treated with exclusive curative intent.

- Group B: 3 patients treated in a postoperative regimen.

- Group C: 6 patients presenting sinonasal tumours.

Thirty-five patients received concurrent chemotherapy (ChT): 16 with CDDP 100 mg/mq, day 1, 22, 43 of radiation treatment, and 19 patients with Cetuximab. In patient receiving Cetuximab, administration was initiated one week before RT at loading dose of 400 mg/mq of body surface area over a period of 120 minutes, followed by weekly 60 minute infusion of 250/mq during RT.

### Volumes definition and dose prescription

A CT scan was performed for each patient with adjacent 3 mm slices. Patients were scanned in supine position, with personalized head mask.

The Gross Tumour Volume (GTV) was defined as the gross extent of tumour shown by imaging, including all involved (positive) lymph nodes. MRI, and in few cases FDG-PET, were used in the delineation of GTV. On the basis of the primary tumour size and involved nodes, the high-risk Clinical Target Volume (CTV1) was defined as GTV (guided by clinical criteria and FDG-PET imaging whenever available) plus a margin for microscopic spread, and the low-risk Clinical Target Volume (CTV2) included precautionally uninvolved nodes. A margin for Planning Target Volume (PTV) was generated by expanding the CTV by 3 mm in all directions except 6 mm in the cranio-caudal direction.

Organs at risk (OAR) were contoured by the planner and included as follow: spinal cord, brain stem, left and right parotids; larynx and uninvolved oral cavity were outlined whenever not heavily included in the target. Whenever close to the PTV, also left and right eyes, optic nerves, and optic chiasm were drawn. In addition, the Healthy Tissue was defined as the patient's volume included in the CT dataset minus all PTV volumes.

Dose was prescribed to mean PTV dose for the high dose level as follows:

- Group A: SIB (Simultaneous Integrated Boost) with two dose levels of 54.45Gy and 69.96Gy in 33 fractions (1.65 and 2.12Gy/fraction, respectively). Six of the 36 patients in this group received a three dose level treatment, with an intermediate level of 59.4Gy (1.8Gy/fraction), of limited volume. In the present study this intermediate target was not analyzed.

- Group B: SIB with two dose levels of 54.45Gy and 66Gy in 33 fractions (1.65 and 2Gy/fraction, respectively).

- Group C: single dose level of 55Gy in 25 fractions (2.2Gy/fraction).

All patients were treated once a day, 5 days a week.

Plans were optimized for one or two isocentric arcs for a Clinac 2100 equipped with a Millennium-120MLC and beam energy of 6MV. Maximum Dose Rate was set to 600MU/min. Further details on RapidArc technique can be found for example in (12,15).

RapidArc plan optimization (with Progressive Resolution Optimizer II implemented in the Eclipse treatment planning system) was performed requiring PTV coverage of 95%-107%. Concerning OARs the objectives were as following: Spinal cord: D_1% _< 46Gy; Brain stem: D_1% _< 54Gy; Parotids (considered separately left and right): V_30Gy _< 45%, D_mean _< 26Gy; Larynx: V_40Gy _< 50%; Oral cavity (not involved): V_40Gy _< 50%; Eyes: V_40Gy _< 50%; Optic nerves and Chiasm: D_1% _< 50Gy. A general strategy was followed during the optimisation process, setting, as priorities, higher values to targets with respect to organs at risk. In addition to the defined organs at risk, a dummy structure drawn as a shell around the targets was used to confine the dose inside the PTV forcing the surrounding healthy tissues to receive lower doses.

All dose distributions were computed with the Anisotropic Analytical Algorithm (AAA, version 8.6) implemented in the Eclipse planning system with a calculation grid resolution of 2.5 mm.

Daily check of patient positioning was performed for all patients by means of kV-cone beam CT (CBCT) system integrated in the machine.

### Data evaluation

Plan quality was analyzed from Dose Volume Histogram (DVH) data.

PTV and CTV (high and low dose levels) coverage was scored through D_2% _(maximum significant dose), D_98% _(minimum significant dose), V_95%_, V_107%_; homogeneity was defined as D_5%_-D_95%_. Dose distribution conformity to PTV was scored as Conformity Index (CI_95%_), defined as the ratio between the patient's volume receiving at least 95% of the dose prescription, and the volume of related PTV; CI_95% _was reported for both high and low dose PTVs. Target data analysis was conducted for each group separately.

Concerning OARs, the mean dose, the maximum dose (as D_1%_) and appropriate values of V_xGy _(volume receiving at least x Gy) were analyzed, but only findings relative to the plan objectives were reported. About Healthy Tissue, similar parameters were analyzed. To account for hot spots, the External Volume Index (EI) was defined as 100*V_D_/V_PTV_, where V_D _is the volume of Healthy Tissue receiving more than the prescribed low dose, and V_PTV _is the volume of all PTV. All dosimetric data were reported as average over all the patients (or patients belonging to a specific group); errors refer to one standard deviation. OARs data of Group A and B were analyzed together; presenting irradiation of similar anatomical regions, while Group C was kept separated involving the sinonasal region only, and not the neck areas.

Technical delivery parameters of RapidArc treatments are reported, as well as the beam-on time (defined without inclusion of patient positioning and imaging procedures).

Results of pre-treatment plan quality assurance are reported as Gamma Agreement Index (GAI), defined as the percentage of modulated field area passing the γ-index criteria with thresholds on dose difference ΔD = 3% of the significant maximum dose, and on Distance to Agreement DTA = 3 mm. Measurements and analysis were performed by means of the GLAaS methodology described in (22,23) based on absorbed dose to water derived from EPID measurements.

### Toxicity evaluation

All patients were evaluated weekly during the RT course and after the completion of the treatment with a predefined follow-up schedule. The here reported data refer to acute toxicity at the end of RT, scored in terms of mucositis, radiation dermitis and dysphagia, according to the Common Terminology Criteria for Adverse Events (CTCAEv3.0) system developed by the National Cancer Institute.

Toxicity data were stratified in Group A+B and Group C due to the different treatment localization, and also for chemotherapy (CDDP, Cetuximab, no chemotherapy) in order to not mix up toxicity coming from the combination of chemo-radiation treatment (e.g. it is known the high skin toxicity when Cetuximab is administered).

## Results

### Dosimetric and technical results

**Figure 1:**
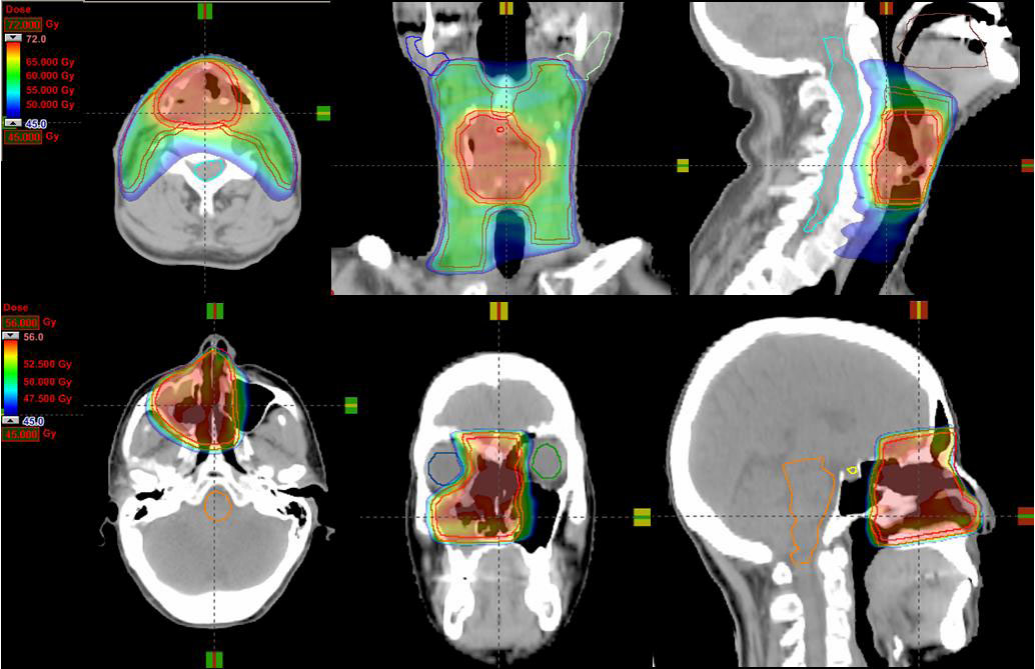
**Dose distributions for two patients (upper row from Group A, lower row from Group C) for axial, coronal and sagittal views**. CTV, PTV, and OARs are outlined.

**Figure 2:**
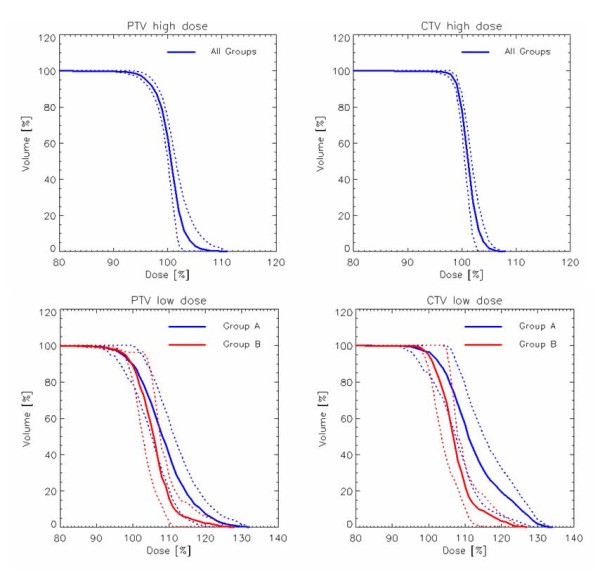
**First row: average (over all patients) DVH for CTV and PTV high dose, with 1SD as dotted lines**. Second row: average (divided for groups A and B) DVH for CTV and PTV low dose, with 1SD as dotted lines.

**Figure 3:**
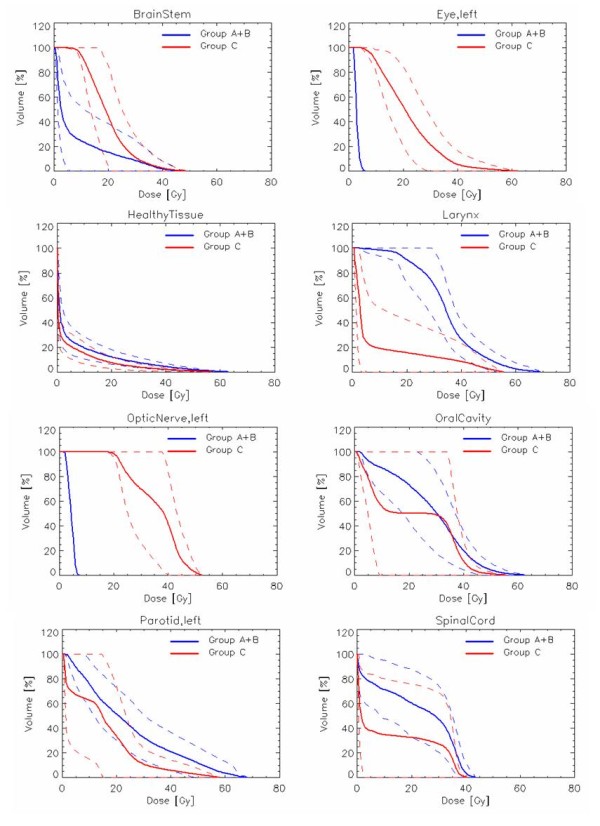
**Average DVHs for OARs, with 1SD as dotted lines, divided for Group A+B and Group C**.

Figure 1 shows examples of dose distributions for one patient of Group A and one patient of Group C in axial, coronal and sagittal views. CTVs, PTVs and main OARs are shown as solid lines. Figure 2 presents the mean DVHs for CTV and PTV stratified as high and low dose (targets of patients of Group C are included in the high dose volumes), while Figure 3 reports mean DVHs for OARs and Healthy Tissue, stratified in Group A+B and Group C. Dotted lines represent inter-patient variability at one standard deviation. In figure 2, second row, a better dose homogeneity in the low dose target (both PTV and CTV) is shown for group B with respect to group A. This variation could be ascribed to the relative difference between the two specific dose levels (being 54.45Gy the low dose, 69.96Gy and 66Gy for the high dose in group A and B, respectively): the larger the difference, the more pronounced the DVH tail to higher doses.

Findings from the DVH analysis are reported in Table 2 for targets (CTV and PTV); while in table 3 OARs and Healthy Tissue are presented, including the specific planning objectives.

**Table 2:** Summary of DVH analysis for PTV

		Objective	Group A	Group B	Group C
PTV high dose	Volume [cm^3^]		142 ± 119	93 ± 68	144 ± 54
	Mean [%]	100%	100.2 ± 0.8	100.5 ± 1.0	100.0 ± 0.2
	D_2% _[%]	< 107%	104.1 ± 2.0	104.0 ± 2.2	104.4 ± 1.7
	D_5-95% _[%]	Minimise	7.2 ± 2.0	7.0 ± 3.4	7.7 ± 2.7
	D_98% _[%]	> 95%	94.6 ± 1.5	91.9 ± 6.5	93.6 ± 2.2
	V_95% _[%]	100	97.2 ± 2.0	98.9 ± 1.7	96.5 ± 2.4
	V_107% _[%]	0	0.7 ± 3.2	0.1 ± 0.2	0.4 ± 0.6
	CI_95%_	1	1.21 ± 0.15	1.48 ± 0.43	1.06 ± 0.06
CTV high dose	Volume [cm^3^]		82 ± 43	59 ± 46	103 ± 41
	Mean [%]	100%	100.9 ± 0.6	101.2 ± 1.3	100.6 ± 0.4
	D_2% _[%]	< 107%	104.0 ± 1.2	104.0 ± 2.1	104.4 ± 1.8
	D_5-95% _[%]	Minimise	4.6 ± 1.6	4.1 ± 1.8	5.6 ± 2.1
	D_98% _[%]	> 95%	97.8 ± 1.1	98.3 ± 1.6	96.6 ± 2.0
	V_95% _[%]	100	99.7 ± 0.7	99.6 ± 0.4	99.2 ± 1.1
	V_107% _[%]	0	0.2 ± 0.5	0.1 ± 0.1	0.4 ± 0.6
PTV low dose	Volume [cm^3^]		253 ± 139	384 ± 282	
	D_98% _[%]	> 95%	95.1 ± 2.4	94.2 ± 2.6	
	V_95% _[%]	100	97.1 ± 5.0	97.8 ± 1.1	
	CI_95%_	1	1.38 ± 0.16	1.55 ± 0.20	
CTV low dose	Volume [cm^3^]		184 ± 105	264 ± 178	
	D_98% _[%]	> 95%	100.7 ± 2.8	99.8 ± 2.1	
	V_95% _[%]	100	99.0 ± 4.0	99.7 ± 0.3	

**Table 3:** Summary of DVH analysis for OARs

	Objective	Group A+B	Group C
		Spinal Cord	
D_1% _[Gy]	46Gy	37.7 ± 6.8 [max 44.2]	22.5 ± 19.8 [max 39.7]
		Brain Stem	
D_1% _[Gy]	54Gy	25.5 ± 13.0 [max 49.4]	30.2 ± 12.4 [max 48.7]
		Parotid	
Volume [cm^3^]		21 ± 7	24 ± 9
Mean [Gy]	< 26Gy	21.5 ± 6.4 [max 38.2]	14.7 ± 10.2 [max 25.9]
V_30Gy _[%]	< 45%	24.4 ± 13.7 [max 64.6]	10.5 ± 11.8 [max 26.7]
		Parotid-PTV	
Volume [cm^3^]		20 ± 7	24 ± 10
Mean [Gy]	< 26Gy	19.7 ± 5.6 [max 36.4]	13.8 ± 9.3 [max 25.0]
V_30Gy _[%]	< 45%	20.4 ± 11.9 [max 61.9]	8.0 ± 8.6 [max 16.8]
		Oral Cavity	
Mean [Gy]		28.3 ± 9.8 [max 40.8]	
V_40Gy _[%]	< 50%	20.0 ± 15.7 [max 43.2]	
		Larynx	
Mean [Gy]		34.9 ± 6.4 [max 44.3]	
V_40Gy _[%]	< 50%	26.6 ± 14.6 [max 42.8]	
		Eyes	
Mean [Gy]			23.5 ± 8.8 [max 43.6]
V_40Gy _[%]	< 50%		10.1 ± 17.5 [max 57.1]
		Optic Nerves	
D_1% _[Gy]	< 50Gy		46.7 ± 6.8 [max 56.3]
		Chiasm	
D_1% _[Gy]	< 50Gy		40.7 ± 9.0 [max 47.4]
		Healthy tissue	
Volume [dm^3^]		12.57 ± 4.56	11.40 ± 6.42
Mean [Gy]		6.4 ± 2.9	4.4 ± 2.2
V_5Gy _[dm^3^]		3.21 ± 1.42	2.36 ± 1.18
V_10Gy _[dm^3^]		2.45 ± 1.12	1.73 ± 1.00
EI_100%_		0.53 ± 1.26	0.80 ± 0.90
DoseInt [Gy dm^3^]		72.87 ± 25.23	42.78 ± 26.02

D_x% _= dose received by the x% of the volume; V_x% _= volume receiving at least x% of the prescribed dose; CI = ratio between the patient volume receiving at least 95% of the prescribed dose and the volume of the total PTV. DoseInt = Integral dose, [Gy cm^3 ^10^3^]

Dosimetric data showed a good sparing of OARs as well as good target coverage, with respect to planning objectives for all the included parameters.

The target volume receiving at least 95% of the prescribed dose is higher than 97% for group A and B, while slightly less for group C, due probably to the higher tissue inhomogeneity in ethmoidal regions (with a lot of small cavities); for all groups the V_95% _mean value of the CTV is higher than 99%.

As regards OARs, the serial organs as spinal cord and brain stem never reached the tolerance level, being the average value of maximum dose well below the tolerance criteria. Concerning parotids, the gland volume included in the PTV was in average 6 ± 8% with a maximum value of 19% (this small overlap is mainly due to the CTV to PTV margin, being only of 3 mm. Moreover it is an internal rule to eventually reduce this margin toward parotids if judged clinically acceptable); one parotid over all glands of all patients was excluded from the analysis having 40% of its volume inside the PTV: in this case it was not considered in the optimisation process to not compromise the target coverage. In average the parotid objectives were largely satisfied, except for three cases, where the mean dose was higher than 30Gy, with only one of those having also V_30Gy _higher than the goal of 45%, being of 50%. In table 3 data for both structures, Parotid and Parotid-PTV are reported for completeness. Oral cavity and larynx for Group A+B fulfilled widely the requested objectives. For optical apparatus (eyes, optic nerves and chiasm) in Group C patients, the goals were achieved except in one case, where the tolerance values exceeded by about 15%.

Concerning Healthy Tissue, a higher dose bath is delivered to Group A+B patients than Group C, due to higher dose prescriptions, and more difficult target shape, with strong concavities, present in the first group. This is confirmed by the higher CI reported for Groups A and B with respect to Group C.

Technical parameters of the treatments are summarized in table 4: more than 70% of the cases were planned with 2 arcs, but keeping the average delivery time below 2 min. Indeed the dose rate was the dose modulating parameter, being well below 600 MU/min (and consequently the gantry speed was at its maximum value of 4.8 degree/sec). In a large portion of cases the arcs were not set as whole rotation (mean arc length was 312 ± 42 degree), also to avoid, in the most posterior entries, the moving rails that are present in the treatment couch, and that were always positioned to their most internal setting.

**Table 4:** Technical characteristics of RapidArc plans

Number of arcs	1 (13), 2 (32)
Arcs length [˚]	312 ± 42
Beam energy	6 MV
Delivery time [min]	1.80 ± 0.62
MU/fraction	458 ± 112
MU/Gy	219 ± 51
Dose Rate [MU/min]	264 ± 88
Gantry speed [deg/sec]	4.8 ± 0.0
Collimator angle [˚]	(±)17 ± 6
Mean leaf aperture [cm]	3.0 ± 0.8
Mean CP area [cm^2^]	44.0 ± 16.8
Mean field area [cm^2^]	219 ± 93
Gamma Agreement Index 3%,3 mm [%]	96.7 ± 2.1 [90.1, 99.7]

MU: monitor units, CP: control point

Pre-treatment quality assurance of RapidArc arcs resulted in an average gamma agreement index GAI of 96.7 ± 2.1%, higher than the acceptance threshold of 95% set as a reference in our institute. In few cases (three with GAI around 93%, one with GAI 90%) this threshold was not achieved, but plans were accepted after careful evaluation of the location of the discrepancies, as well as the measured/calculated dose profiles. The discrepancies were mainly found in the interleaf regions.

### Clinical results

Table 5 reports findings in terms of toxicities. Two patients were not evaluated, because they had unplanned treatment interruption due to rapid worsening of general conditions.

**Table 5:** Acute toxicity

		All	**No chemoth**.	CDDP	Cetuximab
Group	A B C	36 3 6	4 1 5	13 2 1	19 0 0
Completion of RT	Completed Interrupted	43 2	10 0	16 0	17 2
Mucositis	G0 G1 G2 G3	6 17 8 12	5 3 1 1	1 9 4 2	0 5 3 9
Dermitis	G0 G1 G2 G3	6 20 11 6	6 3 1 0	0 13 3 0	0 4 7 6
Dysphagia	G0 G1 G2 G3	10 11 19 3	5 2 2 1	3 6 7 0	2 3 10 2

In the group of the analyzed patients, no grade 4 acute toxicity was observed. The most common acute G3 toxicities were mucositis (28%), followed by dermitis (14%) and dysphagia (7%). Nevertheless, no patients required percutaneous gastrotomy or feedings tubes. Stratifying patients according to chemotherapy modality, patients treated with Cetuximab presented the majority of G3 toxicities not only for mucositis but also for dermitis and dysphagia. To notice is the peak of toxicity for Cetuximab patients, shifted to G2 or G3 (whichever the toxicity), while for CDDP patients the peak is mainly at G1. To consider is the fact that patients receiving Cetuximab had in average a worse performance status at the beginning of RT: mean performance status value of Cetuximab patients was 0.9, with respect to an average of 0.2 of groups receiving CDDP or no chemotherapy. Concerning compliance, 43 of 45 patients completed treatment (treatment interruption occurred in two patients treated with Cetuximab).

Late toxicity was not assessed in this investigation because of short follow-up. Preliminary clinical results are here reported: at first evaluations, after 2 and 6 months, 31 patients were followed, while 30% of the initial 45 patients had not first evaluation. Twenty-three patients presented complete remission (74%), 5 presented partial remission (16%), and 3 presented stable disease (10%), according to WHO of Response Evaluation Criteria in solid Tumors-RECIST-Group. To underline is that 100% of patients who received CDDP presented complete remission, while this occurred to 56% of patients treated with Cetuximab.

## Discussion

The initial experience of the Istituto Clinico Humanitas on RapidArc technology applied to 45 head and neck patients confirmed the findings of good dosimetric results and of toxicity, as well as the reliability and efficacy of the RapidArc modality as anticipated in dosimetric investigations (19-21).

From the dosimetric viewpoint, presenting Group A+B and Group C distinct anatomical locations, the analysis at the level of OARs has been shown separately in order to not confound global results. Avoiding this bias, the general conclusion of a safe sparing of the parotids for Group A+B is supported, being the mean dose was well below the threshold proposed by Eisbruch *et al *(24) of 26Gy (and subsequent studies (4, 5)). This would suggest an acceptable degree of xerostomia with related acceptable quality of life, with good probability of a substantial preservation of the saliva flow rate. Published examples of clinical experience with IMRT, such as e.g. de Arruda *et al *(25), Chao *et al *(26), and Eisbruch *et al *(24), show improvement of this parotid related parameter, with values of 25 ± 4 Gy.

The intensity modulated techniques - fixed gantry fields and modulated arcs - allowed, since their initial appearance, the treatment of SIB with the therapy delivered to various dose levels with the same plan. The increase in dose/fraction for the high dose level does not correspond to an increase of dose to the OARs (27). This opportunity is widely used for head and neck cancers, differentiating the high risk, intermediate (if any), and low risk of recurrence.

The usage of one or two arcs is related especially to the target and patient anatomy complexity. Generally, with SIB approach, the usage of two arcs is preferable, as also pointed out by Verbakel et al (19) to improve target dose homogeneity and Vanetti et al (20) to improve OARs sparing. In our patient population about two third received two arcs. The small extra time needed to re-program the linac for the second arc, and to deliver the second arc (generally less than 74 sec) is anyway largely inferior to the time needed to deliver such treatments with IMRT. For example a dual arc RapidArc treatment takes about three minutes, while a seven fixed gantry IMRT fields (often splitted) takes approximately 15 minutes to be delivered. This, in terms of patient comfort under the fixation mask, is one of the significant advantages in using the RapidArc technology for head and neck patients. With RapidArc treatment those patients can be easily treated with good dose distributions in a time slot of 10 minutes, including also the time needed to perform a good imaging through a 2D-2D matching (with kV-kV or kV-MV images) or a CBCT. At Istituto Clinico Humanitas a CBCT is acquired before every fraction, keeping the time slot of 10 minutes, following specific internal protocol of quality assurance in terms of patient positioning. This procedure gives confidence in patient treatments allowing the usage of rather small CTV to PTV margins. Moreover, in the head and neck region, clinicians can easily detect tumour variations of the patient volume and anatomy.

In terms of planning time, it could be roughly estimated in about one hour for RapidArc (those have not to be considered as definitive time values because they are dependent on planner, usage of pre-defined objective templates, hardware performances), or half an hour for IMRT with fixed gantry entrances. To consider in the frame of time spent to the treatment preparation there is also the pre-treatment QA process that, with respect to IMRT, has for RapidArc a shorter time due to the limited number of arcs or fields to check.

With respect to pre-treatment QA, head and neck plans with RapidArc showed to be reliable in terms of dose calculation, being within tolerance in the majority of the cases. The here shown data are coherent with what reported in literature for the pre-clinical planning studies, where also some findings concerning delivery was reported. For example the study on head and neck cases published by Verbakel et al (19) showed agreement higher than 97% using films and gamma criteria of DTA = 2 mm and ΔD = 3%; Nicolini *et al *(28) reported agreement around 99% for the same criteria adopted in the present study, using both GLAaS method and Seven29 2D-array (PTW) inside the Octavius phantom for RapidArc bilateral breast cases. Concerning the here presented clinical cases, the average GAI of almost 97% demonstrates the robustness of the delivery in a clinical environment. As described in the Result section, the few cases out of the acceptability level were deeply and critically analyzed and understood before treating the patients. To notice is the locations of the failing points, being in the interleaf spaces, where the high resolution of the detector (EPID) emphasizes the difficulties of the treatment planning system in properly manage the interleaf leakage and tongue and groove effect. It is also for this reason that the collimator is rotated during the arc, in order to smear the effect inside the patient without causing any valuable effect in terms of dose distribution.

On the clinical side data are here reported on acute side effects for patients treated with RapidArc with or without chemotherapy, and on early and preliminary results on local control.

All patients completed RT treatment, except two cases that started in bad general conditions at enrolment; evaluating concomitant chemo-radiotherapy, all patients received the planned number of chemotherapy cycles. Only in the group of patients receiving Cetuximab there were treatment breaks, but not longer than one week. Low grade of mucositis and dermitis, and moderate grade of dysphagia were the most prevalent acute toxicities, whereas mucositis severe enough to necessitate gastrotomy or feeding tube were not present. Data regarding the prevalent toxicities in Cetuximab's patients must be read considering that patients with a low performance status or unfit were enrolled to receive Cetuximab.

Often high grade acute toxicities do not allow completing the planned concomitant treatment. With the advent of IMRT, the possibility to obtain highly conformal dose distributions around the tumour volume, while sparing the nearby sensitive structures has greatly improved. The question of whether this dosimetric improvement creates less acute toxicities remains open. By our experience, RapidArc is able to determine low grade acute side effects and permits to associate concomitant chemotherapy. Excluding dose painting impact of IMRT, in reducing OAR involvements as well as acute toxicities, another possible reason of high tolerability in our patient population could be ascribed to the intense frequency of clinical controls during treatments. It is an our policy to check patients more than once per week in order to detect acute side effects as early as possible and prescribe personalized supportive care during radio-chemotherapy, also avoiding or minimizing interruptions.

## Conclusion

Forty-five patients presenting head and neck cancer were treated with Volumetric Modulated Arc Therapy according to the RapidArc implementation at Istituto Clinico Humanitas during 2009. Quality of treatments resulted in a general fulfilment of planning objectives. Clinical outcome for early acute toxicity showed, as expected, higher toxicity levels for skin and mucosa reactions in patients receiving concomitant Cetuximab chemotherapy. Future investigations will aim to assess at long term definitive outcome, having this first phase achieved the primary goal to demonstrate safety and efficacy of RapidArc.

## Competing interests

Dr. L. Cozzi acts as Scientific Advisor to Varian Medical Systems and is Head of Research and Technological Development to Oncology Institute of Southern Switzerland, Bellinzona.

## Authors' contributions

MS and AF coordinated the entire study. Patient accrual and clinical data collection was done by MS, SC, CB, MB, PN, SP. Data analysis, physics data and treatment planning data collection was conducted by AF, PM; clinical data collection was conducted by MS, CB, MB. The manuscript was prepared by AF. All authors read and approved the final manuscript.
